# Adsorption of Mixed Micelles of Polysorbate 80 and Oleic Acid to the Air–Water Interface

**DOI:** 10.1021/acs.langmuir.5c05532

**Published:** 2026-01-05

**Authors:** Nooshin Sadat Ayati, Ankit D. Kanthe, Mary Krause, Songyan Zheng, Honghu Zhang, Luis E. Ortuno Macias, Charles Maldarelli, Raymond S. Tu

**Affiliations:** † Department of Chemical Engineering, 14770 City College of New York, New York, New York 10031, United States; ‡ Drug Product Development, 3971 Bristol Myers Squibb, New Brunswick, New Jersey 08901, United States; § National Synchrotron Light Source II, 8099 Brookhaven National Laboratory, Upton, New York 11973, United States

## Abstract

The solubilization of long chain amphiphiles with limited solubility into mixed micelles composed of highly soluble surfactants plays a crucial role in modulating the stability and functionality of formulations in pharmaceutical and food systems. Subsequently, the mixed micelles adsorb from solution to the air–water surface, defining the transport mechanism of the insoluble amphiphiles to the interface. We use X-ray reflectivity to measure the composition and provide insight into the structure of these mixed monolayers, and, alongside interfacial tension measurements, we provide an understanding of how the bulk composition determines the surface composition and the dynamics of tension reduction. We use a model system consisting of an insoluble fatty acid, oleic acid (OA), and a soluble micelle-forming surfactant, polysorbate 80 (PS80). PS80 forms spherical micelles, and, above the critical micelle concentration (cmc), OA is readily solubilized inside the micelles. We show that the adsorption of PS80/OA mixed micelles to the air–water interface rapidly reduces the tension and lowers the equilibrium tension in proportion to the OA concentration. X-ray reflectivity data, fit using Parratt-slab models, quantitively demonstrates that the monolayers become enriched with OA, and we show how the OA intercalates into the PS80 monolayers.

## Graphical abstract

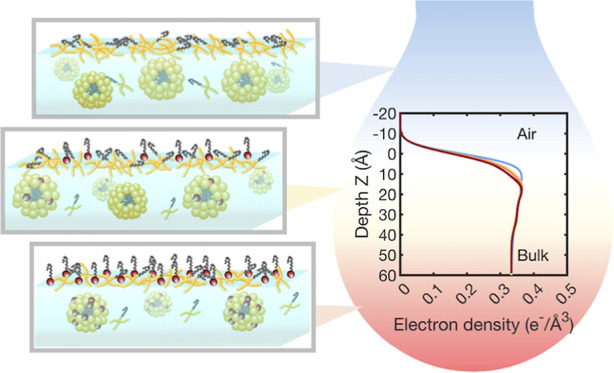

## Introduction

At sufficiently high bulk concentrations, surfactants can self-assemble into micelles.^1^ Depending on their molecular geometry, amphiphiles organize into nanoscale oil-like domains in water, defining the critical micelle concentration (cmc) at which micellization begins.^2–4^ Below the cmc, dissolved surfactant monomer adsorbs to the air–water interface where they form monomolecular interfacial layers. Above the cmc, adsorption is augmented as both the monomers and micelles adsorb to the air–water interface. Micelle adsorption and subsequent unzipping or disassembly at the sublayer accelerates the kinetics of adsorption,^5^ leading to faster surface tension reduction. When a long-chain insoluble amphiphile is added to the micelle solution, the hydrophobic molecules with low solubility in aqueous phases can partition into the oil-like micellar domains, forming mixed micelles that dramatically increase the “solubility” of the insoluble amphiphile.^6^

This two-component colloidal principle-mixed micelles composed of soluble and insoluble species-underlies numerous natural and engineered processes. In the physiological process of digestion, lipolytic enzymes in bile digest oil drops into poorly soluble amphiphiles such as long-chain fatty acids, which are then solubilized in bile salt micelles.^7–9^ Similarly, in pharmaceutical formulations where many active ingredients are hydrophobic, solubilization within micelles provides a key route for oral bioavailability and therapeutic administration.^10–17^ In chemical synthesis, catalytic reactions in organic media can be realized in water by arranging the poorly water-soluble reactants in the hydrophobic domains of aqueous micellar solutions.^18^ This micellar catalysis represents a sustainable, green chemistry approach to avoid the large-scale use of organic solvents.

Prior work has explored the solubilization of insoluble surfactants in aqueous micellar solutions, focusing on the solution phase behavior (e.g., solubility limits, transformations of micelle form with solubilization^19^). Our work will examine the interfacial behavior, focusing on the role of mixed micelles as the key mechanism that populates the air–water interface with both the insoluble and soluble surfactants. Upon adsorption, the insoluble amphiphile (e.g., a long chain alcohol or a protonated fatty acid such as oleic acid (OA)) is transferred to the surface alongside the soluble surfactant, forming a mixed interfacial monolayer whose structure and properties (e.g., surface tension and stability) depend on the relative composition and packing of the two species in the bulk solution. These properties can be markedly different from the surfactant-only layers.^9,20–23^ Foams generated from these solutions have been found to be highly stable as the reduction of interfacial mobility reduces the gravity driven drainage and coalescence of the foam. Industries relying on foams with extended lifetimes (e.g., food and personal care industries) use these long-chain insoluble amphiphiles for foam stabilization.^24–26^

In the pharmaceutical industry, surfactants are added to formulations of therapeutic biologics (e.g., monoclonal antibodies) to prevent the adsorption of the therapeutic product to air–water interfaces introduced during storage or administration. The surfactants—typically polysorbates with ethylene oxide polar groups (Tween 20 or PS80)^27–29^ —are present above micellar concentrations. These surfactants adsorb more rapidly to the surface than the protein and form a protective interfacial layer in which the ethylene oxide chains hinder further protein adsorption.^30^ However, polysorbates are susceptible to enzymatic degradation, generating free fatty acids such as OA. OA, an insoluble degradation product,^31,32^ can be solubilized within micelles and delivered to the surface, forming a modified layer that no longer protects the surface from proteins—potentially leading to aggregation, particle formation, and loss of drug potency.^33–39^

In this work, we examine the macroscopic properties of mixed interfacial layers formed by adsorption from micellar solutions, particularly with respect to surface tension and stability.^23–25^ The composition of the mixed layer at the air–water interface relative to that in the bulk phase remains unknown, limiting our ability to establish quantitative structure–property relationships between bulk formulation and interfacial performance. To address this gap, our study explores PS80 as the soluble surfactant and OA as the insoluble amphiphile in our model system. The incorporation of OA into PS80 micelles bypasses its solubility barrier,^24^ making this an ideal system for investigating how micelle composition affects interfacial adsorption. Our aim is to use X-ray reflectivity, which has previously been used to measure the surface concentrations of multicomponent systems at an air–water interface,^30,34^ to quantitatively measure the composition of the mixed interfacial layer obtained by adsorption of PS80/OA mixed micelles.

## Materials and Methods

### Materials

Polysorbate 80 (average molecular weight 1310 g/mol) and histidine are donated by Bristol-Myers Squibb, New Brunswick, NJ, and were used as received. NaOH for pH adjustments (purity >99%) is obtained from Fisher Scientific. Oleic acid (purity >99%, Sigma-Aldrich) was dissolved in chloroform (purity >99.5%, Sigma-Aldrich) for pure OA spreading experiments at the air–water interface. PS80 and mixed PS80 with OA solutions are prepared from the stock solution of PS80 in 20 mM histidine buffer with pH 6. The stock solutions and the buffer are filtered using 0.22 μm polyvinylidene fluoride (PVDF) filters. Ultrapure water for dilution of samples is obtained from a Mili-Q water filtration unit (EMD Millipore) with a resistivity of 18.2 MΩcm. The fluorescence dye Oil Red O obtained from Sigma-Aldrich, was dissolved in Isopropanol 0.5% W/V and added directly to the PS80 and OA solutions (4% v/v).

### Surface Pressure Measurements

Surface pressure–area compression isotherms were recorded using a KSV Teflon Mini trough (Biolin) at room temperature. The trough with Delrin barriers has a width of 5 cm and a maximum area of 8850 mm^2^. To measure surface pressure, Wilhelmy plates made of Whatman filter paper were employed. Prior to use, the trough and barriers were carefully rinsed with ultrapure water, followed by ethanol, and then rinsed once more with ultrapure water. They were finally wiped with Kimwipes (Fisher) to ensure cleanliness. Monolayers were prepared by spreading a 0.25 mg/mL oleic acid solution in chloroform onto the DI water subphase using a 25 μL Hamilton syringe. All experiments were conducted at room temperature (23 ± 0.5 °C). After spreading, the solvent was allowed to evaporate for 30 min, ensuring complete evaporation before the compression step. Compression of the monolayer was carried out at a constant rate of 10 mm/min. To verify the reliability of the results, each isotherm was measured at least three times to confirm reproducibility.

### Dynamic Tension Measurements

Dynamic surface tensions at the air–water interface were measured using a pendant bubble tensiometer (Attension Theta, Biolin Scientific, Stockholm, Sweden). To prepare the measurements, solutions with varying concentrations of PS80 and OA were made, and 12 μL droplets were generated and suspended from an 18-gauge metal needle. The contour of the hanging droplet was captured through an optical system, with images recorded by a camera. Surface tension was calculated by fitting the droplet’s outline to the Young–Laplace equation, which describes the relationship between the droplet shape and its surface tension. This setup allowed precise determination of the dynamic surface tension as a function of time.

To measure dynamic changes in surface tension, the droplet was rapidly formed, allowing PS80 and OA to compete for adsorption at the air–water interface. The dynamic tension was obtained from images of the droplet relaxation over a 10,000 s period at a frame rate of 6.5 fps. Before starting each experiment, the syringe, needle, and all connections were thoroughly cleaned with deionized (DI) water and sonicated for 1 h to ensure no contamination. Experiments were conducted at room temperature (23 ± 0.5 °C). To establish a baseline surface tension of 72.5 ± 0.3 mN/m, the surface tension of the clean air–water interface was first measured using DI water.

### Liquid Surface X-ray Reflectivity Measurements

X-ray reflectivity (XRR) measurements were conducted at the Open Platform and Liquid Scattering (OPLS) end station of the Soft Matter Interfaces (SMI) beamline (12-ID) at the National Synchrotron Light Source II, Brookhaven National Laboratory. Liquid samples, consisting of varying concentrations of PS80 and oleic acid (OA), were prepared in three parallel Teflon troughs, each with a maximum surface area of 40.3 cm^2^ and containing approximately 18 mL of solution. These troughs were placed in a sealed aluminum box mounted on a vibration isolation table, with the internal atmosphere maintained at an overpressure of water-saturated helium to minimize background scattering. Prior to X-ray measurements, the samples were allowed to equilibrate with equilibration times determined by the pendant drop tension equilibration times. All measurements were performed at room temperature.

For the OA deposition experiment, the same trough was used for all sample depositions to ensure consistent deposition times across all samples. Monolayers were prepared by spreading a selected volume of 0.25 mg/mL oleic acid solution in chloroform onto the DI water subphase using a 25 μL Hamilton syringe to achieve the target surface concentration. A 15 min waiting period was observed before each experiment to ensure complete evaporation of the chloroform solvent. The sample chamber was purged with helium to reduce the oxygen content to below 1%, followed by a 40 min scan of the surface of each trough. XRR data were collected over a *Q*_*z*_ range of 0.016 Å^–1^ < *Q*_*z*_ < 0.6 Å^–1^, where *Q*_*z*_ is the wave vector transfer along the surface normal, calculated as *Q*_*z*_ = (4π/λ) sin­(α), with a wavelength of λ = 1.28 Å. To mitigate radiation damage, the incident beam was shifted across different regions of the sample’s interface.

The analysis of XRR data involved modeling the interfacial electron density profile ρ­(*z*) using XModFit software.^40^ The electron density profile was fitted using a model based on sum of error functions, and the data were normalized to the Fresnel reflectivity (RF) for a theoretical liquid–air interface. The model function used to describe the electron density profile (EDP) is given by

$$\rho \left(z\right)=\frac{1}{2}\sum_{i=0}^{N-1}\mathrm{erf}\left(\frac{z-z_{i}}{\sqrt{2}\sigma }\right)\left(\rho_{i}-\rho_{i+1}\right)+\frac{\rho_{0}-\rho_{N}}{2}$$

where $\mathrm{erf}\left(z\right)=\left(\frac{2}{\sqrt{\pi }}\right)\int_{0}^{\pi }e^{-t^{2}}dt$ is the error function, *N* is the number of internal interfaces in the surface film, σ is the interfacial roughness, ρ_0_ the electron density of the aqueous phase, (*z* – *z*_*i*_) is the thickness of *i*th slab (*d*_*i*_), ρ_N_ the electron density of the air phase, and ρ_*i*_ and *z*_*i*_ represent the electron density and position of the *i*th slab, respectively. The resulting profile allowed for the determination of structural parameters such as the thickness, interfacial roughness, and electron density of the surface layers. The layer thickness (*d_i_*) determines the extent of a given electron-density region into the aqueous phase. The electron density (ρ*_i_*) sets the magnitude of contrast relative to the surrounding medium, defining how strongly each layer contributes to the overall scattering. The interfacial roughness (σ) arises from capillary-wave broadening and the intrinsic gradient in electron density between adjacent layers. In the model function, σ enters through the error-function smoothing term, so larger σ values produce more gradual transitions between layers rather than abrupt steps. Together, these parameters define the shape of the EDP and determine the structural interpretation of the interfacial film. Though this approach, the effects of oleic acid incorporation into PS80 micelles at the air–water interface were characterized, providing insights into the composition and structure of the interfacial layer.^41–44^ Despite similar reflectivity curves, X-ray reflectivity (XRR) resolves angstrom-scale variations in interfacial electron density that cannot be distinguished by surface tension measurements. The analysis is constrained by physically meaningful models, and the resulting structural differences are systematic and reproducible across measurements. Parameter uncertainties were estimated using Markov Chain Monte Carlo (MCMC) sampling and the reported errors correspond to the 84% credible interval of the posterior distributions. For conservative estimation, the reported error is the larger of the two bounds from the asymmetric interval. In addition, standard analytical error propagation for independent variables was used to calculate the uncertainties in surface concentrations and ratios.

### Dynamic Light Scattering

The measurements were conducted using a Zetasizer Nano ZS (Malvern) at 23 ± 0.5 °C. A 1 mL sample of each solution containing varying concentrations of PS80 and OA was analyzed in plastic cuvettes. The particle size distribution, expressed as volume percentage, was obtained using the instrument’s regularized (CONTIN-type) algorithm, and hydrodynamic diameters were calculated from the diffusion coefficients using the Stokes–Einstein equation.

### Confocal Laser Scanning Microscopy (CLSM)

The samples were imaged using a Leica TCS SP2 AOBS confocal microscope equipped with argon-ion and HeNe lasers. A 63×/1.4 NA oil-immersion objective was used for all CLSM images. For Red Oil O, excitation was performed using the 561 nm laser line at 2% intensity, and fluorescence was collected with a detection window between 580 and 700 nm. Images were acquired in a 1024 × 1024-pixel format at a scan speed of 2600 Hz, covering a total z-range of 950 nm. The pinhole aperture was set to an Airy unit of 1.0. Summed z-projections of the OA droplet stacks were generated using ImageJ (version 1.54g).

Liquid samples were mounted using a thin, coverslip-based chamber geometry. Two high-precision No. 1 borosilicate coverslips (0.13–0.17 mm thickness; Fisher Scientific, 18 × 18 mm) were placed parallel to each other, and approximately 10 μL of solution was introduced into the narrow gap between them by gently pipetting at the edge. The liquid spread across the chamber, forming a uniform layer comparable to standard cell-culture chamber slides. Imaging was performed immediately after loading (<3–5 min) to minimize evaporation.

### Cryogenic Electron Microscopy (Cryo-EM)

Cryo-electron microscopy (cryo-EM) grids were prepared using lacey carbon support films with an ultrathin carbon layer (<3 nm) on copper grids (Ted Pella, Inc., product #01824). Prior to sample application, all grids were glow-discharged for 20 s in a Fischione Nanoclean 1070 (70% power) using a 75:25 mixture of argon and oxygen. A 3 μL aliquot of the sample solution was applied to the freshly treated grids, which were then incubated for 60 s. Grids were blotted with filter paper (blot force 4, blot time 3.5 s) in a Vitrobot Mark IV operated at 21 °C and 100% relative humidity, followed by rapid vitrification by plunging into liquid ethane precooled by liquid nitrogen. Prepared grids were stored in liquid nitrogen until imaging.

For imaging, cryo-EM grids were transferred under liquid nitrogen to a Gatan 626 cryo-specimen holder and maintained at −170 °C during data collection. Imaging was performed in low-dose mode on a Titan Halo transmission electron microscope (Thermo Fisher Scientific) operating at 300 kV and equipped with a CETA camera. A defocus of −3 to −5 μm was applied to enhance image contrast with minimal loss of resolution, consistent with standard cryo-EM practice. All features in each image lie within the same nominal defocus range, with variations of only tens to ∼100 nm due to the allowable ice thickness for vitrified samples.

## Results and Discussion

### Spread Monolayers of Oleic Acid

We begin with a study of spread monolayers of oleic acid. Figure 1c presents the surface pressure isotherms for 0.25 mg/mL OA solution in chloroform spread over pure water, presenting surface pressure (Π) as a function of molecular area (*A*). The compression isotherm for OA on water is in good agreement with previous studies on OA monolayers.^45,46^ The error bars represent the variability observed across three replicate experiments, and fresh spreading solutions were used to assess reproducibility. The OA surface pressure area isotherm indicates a more compressible monolayer than the straight chain analogue, stearic acid which forms liquid condensed (LC) and solid states (LS),^47,48^ with the OA layer remaining in a liquid expanded (LE) state due to the cis-bond that prevents chain stacking to more condensed states. OA monolayer collapses at 28 mN/m according to the literature.^45,49,50^ However, in our experiments we did not compress to the collapse point, as our focus was on the liquid-expanded regime.

**Figure 1:**
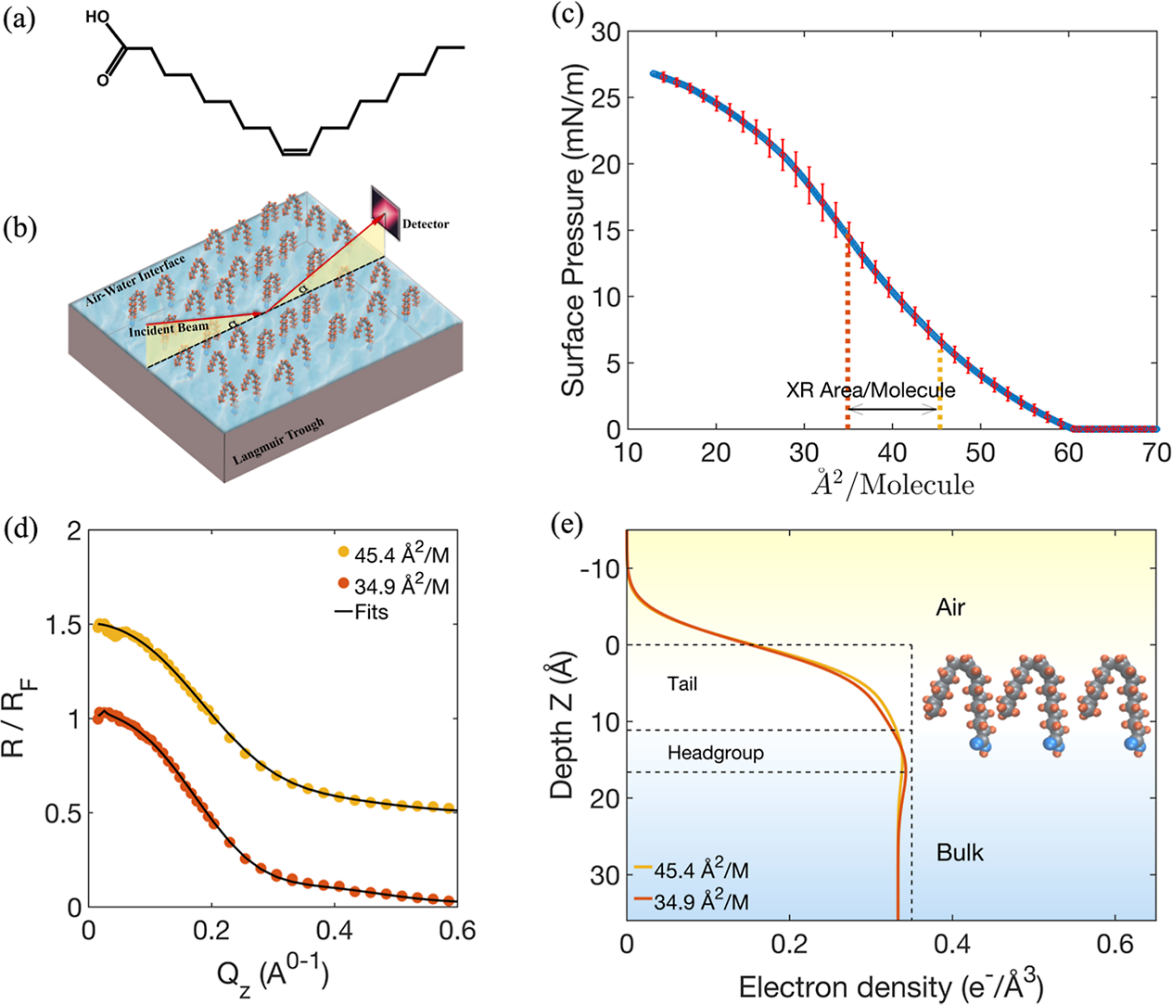
(a) Chemical structure of OA, (b) schematic of the X-ray reflectivity measurement, (c) surface pressure–area isotherm for oleic acid monolayer on water, error bars represent the standard deviation from three independent measurements, (d) normalized reflectivity (*R*/*R*_F_) of XRR measurements from adsorbed layers of OA at the air–water interface as a function of *Q*_*z*_ normal to the surface at 45.4 and 34.9 Å^2^/molecule, the upper XRR curve is shifted by 0.5 for clarity, (e) EDP as a function of the distance *z* normal to the surface of OA at 45.4 and 34.9 Å^2^/molecule.

Following the surface pressure measurements Figure 1c, we employed X-ray reflectivity to determine the detailed structure and composition of the oleic acid monolayer at the air–water interface, Figure 1b. Two specific surface pressures within the liquid-expanded region of the pressure–area isotherm were selected, representing molecular areas of 45.4 Å^2^/molecule and 34.9 Å^2^/molecule, which translate to OA concentrations of 0.1 μg/cm^2^ and 0.13 μg/cm^2^, respectively. To achieve these concentrations, a monolayer of OA was deposited on the air–water interface from a chloroform solution, and X-ray reflectivity measurements were performed. The resulting reflectivity (*R*) as a function of wave vector transfer (*Q*_*z*_) is shown in Figure 1d, with the specular reflection data fitted to slab models to derive the electron density distribution across the interfacial layer, as depicted in Figure 1e. In the slab model, the interfacial region is divided into distinct layers, each characterized by its own thickness and electron density (Table 1). In the two-slab model for OA, slab No. 1, located in the air phase above the surface, contains only the hydrophobic tail of the oleic acid molecules. Slab No. 2 represents the headgroup of oleic acid along with the surrounding water molecules. The analysis (see Supporting Information) indicates that increasing the OA concentration from 0.1 μg/cm^2^ to 0.13 μg/cm^2^ leads to a narrowing of the reflectivity curve in *Q*_*z*_ space. This is indicative of the formation of an adsorbed fatty acid monolayer.

**Table 1:** Fitting Parameters from XR Measurements from Adsorbed Layers of Oleic Acid Monolayer Spread over the Water Subphase^a^

$$A_{XRR}\left(\frac{{Å}^{2}}{molecule}\right)$$	slab number	*d* (Å)	ρ $\left(\frac{e}{{Å}^{3}}\right)$	σ (Å)
45.4	1	10.63 ± 0.0407	0.312 ± 0.000061	3.594 ± 0.0031
	2	5.025 ± 0.0519	0.350 ± 0.00033	3.594 ± 0.0031
34.93	1	11.64 ± 0.175	0.303 ± 0.00098	3.758 ± 0.0217
	2	6 ± 0.357	0.357 ± 0.00195	3.758 ± 0.0217

a Parameters: *A*_XRR_ is the surface area of deposited OA, *d* is the layer thickness, ρ is the density, and σ is the interfacial roughness, uncertainties reflect MCMC of XRR fitted parameters.

The surface concentration of OA molecules in the monolayer on the water substrate was calculated using a molar volume balance equation and a total electron density balance (see Supporting Information). Table 2 summarizes the calculated surface concentrations derived from the electron density profile. The results show that the area (Å^2^) per molecule for different OA deposition concentrations aligns closely with the selected values derived from the surface pressure–area isotherm shown in Figure 1c. Additionally, the analysis indicates the average number of water molecules, 5.3 ± 0.94, that are associated with each carboxylic headgroup, providing a reference for future calculations of the mixed OA/PS80 system.

**Table 2:** Summary of X-ray Reflectivity Measurements of Surface Concentrations for Oleic Acid Monolayer Spread over Water Subphase^a^

*A*_XRR_ (Å^2^/molecule)	*A*_S_ (Å^2^/molecule)	Γ_OA_ (mg/m^2^)	Γ_W_ (mg/m^2^)	*N*_W_
45.4	40.70 ± 0.85	1.15 ± 0.02	0.35 ± 0.02	4.8 ± 0.3
34.9	38.19 ± 4.77	1.23 ± 0.15	0.46 ± 0.13	5.8 ± 1.8

a Parameters: *A*_XRR_ is the surface area of deposited OA, *A*_s_ is the surface area calculated from EDP, Γ_OA_ is the surface concentration of oleic acid from *A*_s_, Γ_W_ is the surface concentration of water, *N*_W_ is the number of water molecules per oleic acid headgroup, uncertainties reflect standard error propagation from MCMC-derived XRR fitted parameters.

### Polysorbate 80 (PS80) Adsorption Layers from Solution

The dynamic surface tension for the single-component adsorption of Polysorbate 80 (PS80) was measured at a clean air–water interface using the pendant drop technique Figure 2b. This method was used to monitor the decrease in surface tension over time as PS80 molecules adsorbed from the bulk solution onto the interface. These single-component measurements provide essential reference curves, with bulk concentrations ranging from 0.0012 to 0.024 mM. This data is critical for understanding the baseline behavior of PS80 and will serve as a comparison for subsequent competitive adsorption studies in mixed PS80 and OA solutions. As the bulk concentration increases, the tension relaxes more quickly to equilibrium as the rates of diffusive transport fluxes of surfactant to the interface increase, and the equilibrium tension is reduced, Figure 2c.

**Figure 2:**
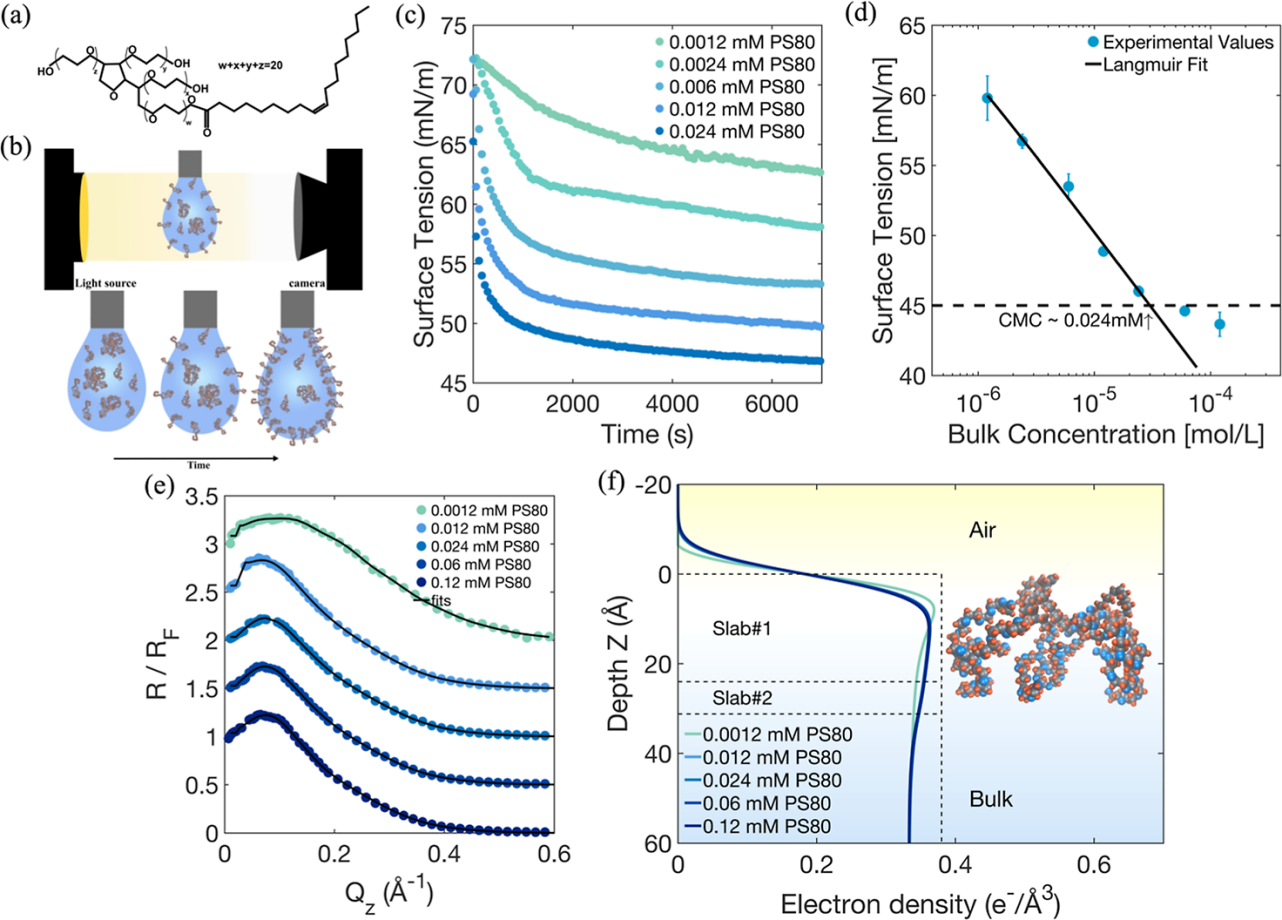
(a) Chemical structure of PS80, (b) scheme of the pendant drop technique, (c) dynamic surface tension of pure PS80 in buffer with concentration range from 0.0012 to 0.024 mM, (d) Langmuir adsorption isotherm fit to the equilibrium surface tension data of PS80 obtained from the pendant drop tensiometry, error bars represent the standard deviation from three independent measurements, (e) normalized reflectivity (*R*/*R*_f_) of XRR measurements from adsorbed layers PS80 at the air–water interface as a function of *Q*_*z*_ normal to the surface at different bulk concentrations of PS80 (0.0012, 0.012, 0.024, 0.06, 0.12 mM), the upper four XRR curves are shifted by 0.5 for clarity, (f) EDP as a function of the distance *z* normal to the surface of different bulk concentrations of PS80 (0.0012, 0.012, 0.024, 0.06, 0.12 mM).

A plot of the equilibrium surface tension, derived from the plateau values observed at long time, as a function of the logarithmic bulk concentration is presented in Figure 2d. From this data, the critical micelle concentration (cmc) of PS80 is determined to be approximately 0.024 mM (within the range reported in the literature^51,52^ ), as the equilibrium surface tension becomes independent of bulk concentration at higher levels. The total surface concentration of the amphiphile can be calculated by analyzing the relationship between the equilibrium surface tension and changes in the bulk concentration of the surfactant.^53,54^ The data are fitted to a Langmuir adsorption isotherm to estimate the maximum surface coverage Γ_∞_ = 2.54 mg/m^2^ (or 1.94 × 10^–6^ mol/m^2^) and the adsorption constant *a* = 1.058 × 10^–7^ mol/L (see Supporting Information). These values are consistent with previously reported values for PS80 in the literature.^30,55^

X-ray reflectivity data for adsorbed layers of pure Polysorbate 80 (PS80) are presented in Figure 2e. Using the instrumental setup in Figure 1b, measurements were conducted at various bulk concentrations of PS80 (0.0012, 0.012, 0.024, 0.06, and 0.12 mM). The data demonstrate a narrowing of the reflectivity curve as the concentration increases from 0.0012 mM to 0.012 mM. Beyond the 0.024 mM threshold, the reflectivity curves converge, indicating that further increases in concentration do not lead to significant changes in the interfacial concentration or structure. This observation is consistent with the surface tension data, where tension reduction follows a similar trend and becomes constant once the PS80 concentration exceeds the cmc, 0.024 mM.

The corresponding electron density profile (EDP) curves, shown in Figure 2f, offer more detailed insights into the structural changes with increased adsorption. By fitting the data to a slab model, the thickness and electron density of the interfacial layer were determined, as summarized in Table S2 (Supporting Information). The results indicate a noticeable increase in electron density from 0.0012 mM to 0.012 mM, suggesting that more surfactant is adsorbing at the interface. However, beyond 0.024 mM, the electron density plateaus, signaling that the PS80 has reached its maximum surface coverage, and no further noticeable increase in density is observed. Based on our fitted 2-layer model (Table S2, Supporting Information) and the electron density profile shown as the dashed line in Figure 2f, the configuration of PS80 at the air–water interface suggests that the hydrophobic tail groups are buried within the interfacial region. The first fitted layer represents both the tail groups and a significant portion of the headgroups, while the second layer corresponds to the terminal ethoxylate chains extending further into the aqueous phase. This compact, interfacial arrangement is consistent with molecular dynamics simulations reported in the literature, which show Tween-80 tails collapsing onto the interface rather than extending into the air and resulting in a condensed, tightly packed monolayer.^56–58^

Similar to the calculation of oleic acid surface concentration, electron density and molar volume balances were applied on PS80 monolayers (see Supporting Information). The surface concentrations derived from the PS80 electron density profile are summarized in Table 3. Once the concentration reaches 0.024 mM, the maximum surface concentration determined from X-ray reflectivity data is approximately 1.8 mg/m^2^. This value is in approximate agreement with the surface coverage predicted by the Langmuir adsorption isotherm fit (Γ_∞_ = 2.54 mg/m^2^). At lower PS80 concentrations, the system equilibrates more slowly, and the X-ray measurements may not capture the same equilibrium state as the surface tension data, since the minimum measurement time is 900 s. This likely accounts for the observed differences in maximum surface coverage. At higher PS80 concentrations, however, the surface tension data indicate that the system has reached equilibrium. In contrast to oleic acid, which carries 5.3 water molecules per oleic acid molecule, the average number of water molecules associated with each PS80 headgroup is calculated to be 69.3 ± 14.46, highlighting the substantial hydration around the polyoxyethylene (POE) groups and providing further insight into the interfacial structure of PS80 at the air–water interface. Characterizing this hydrated structure is essential for understanding how polysorbates interact with hydrophobic residues on protein surfaces and potentially mitigate aggregation.^27,59,60^

**Table 3:** Summary of X-ray Reflectivity Measurements of Surface Concentrations for Polysorbate 80^a^

*C*_PS80_ (mM)	$\Gamma_{PS80}\left(\frac{mg}{m^{2}}\right)$	$\Gamma_{W}\left(\frac{mg}{m^{2}}\right)$	*N*_W_
0.0012	1.30 ± 0.06	1.31 ± 0.06	73.80 ± 4.49
0.012	1.71 ± 0.63	1.72 ± 0.57	73.38 ± 36.52
0.024	1.79 ± 0.72	1.63 ± 0.65	66.20 ± 37.59
0.06	1.86 ± 0.63	1.66 ± 0.57	64.81 ± 31.37
0.12	1.78 ± 0.72	1.67 ± 0.65	68.43 ± 38.42

a Parameters: *C*_PS80_ is the polysorbate 80 bulk concentration, Γ_PS80_ is the surface concentration of PS80, Γ_W_ is the surface concentration of water, *N*_W_ is the number of water molecules per PS80 headgroup, uncertainties reflect standard error propagation from MCMC-derived XRR parameters.

### Competitive Adsorption of PS80 and OA

To investigate the competitive adsorption behavior of PS80 and OA, we began with a constant bulk concentration of PS80 of 0.012 mM, while varying the OA concentrations, 0.017, 0.035, 0.071, and 0.106 mM. After thorough mixing with PS80, the OA mixtures yielded a homogeneous transparent solution, and the pH of all samples remained constant at 6.0. Figure 3a presents dynamic light scattering (DLS) measurements showing the volume percentage as a function of particle size. At lower PS80 concentrations, a relatively small number of micelles exist (Figure S1), and OA addition reduces the effective cmc. As a result, all PS80/OA samples displayed similar profiles, with a dominant peak around 10 nm, indicative of the PS80 micelle size. However, as the OA concentration increased to 0.106 and 0.142 mM, additional peaks at 25 and 300 nm were observed. As the available micelles become saturated by OA, they swell (peak at 25 nm), and excess OA leads to droplet formation, observed as a peak at 300 nm. As the OA concentration increases, not all OA molecules can be accommodated within the micelles, leading to the formation of OA droplets. Oleic acid was studied at pH 6.0; under these conditions, OA is observed to form droplets in our system, as confirmed by confocal microscopy (Figure 3b).

**Figure 3:**
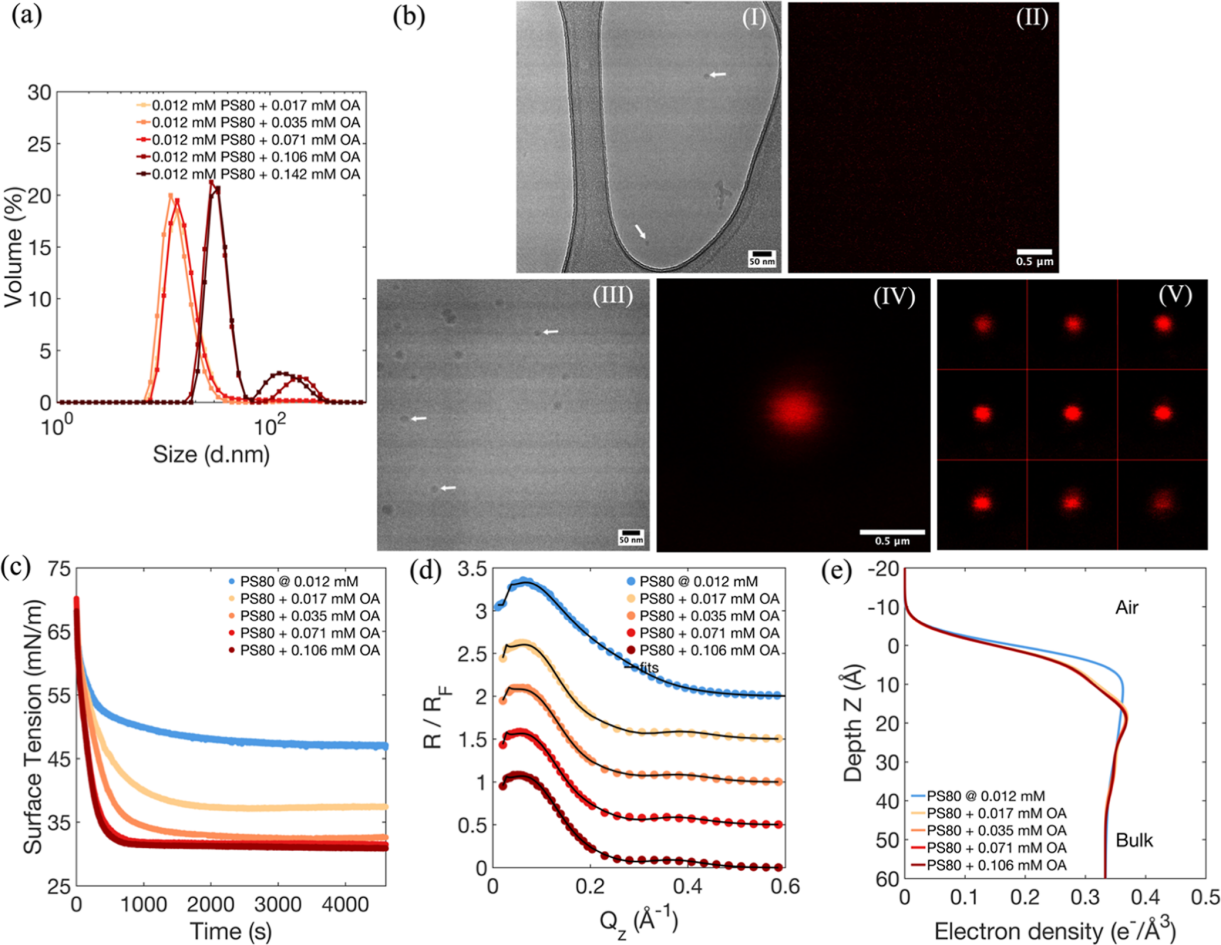
(a) Dynamic light scattering volume distribution as a function of size for 0.012 mM PS80 with different concentrations of OA (0.017, 0.035, 0.071, 0.106, and 0.142 mM), (b) panel I: Cryo-EM images of micelles formed at 0.012 mM PS80, panels II: confocal 3D projections of the 0.012 mM PS80 sample obtained from summed Z-projections of the CLSM Z stacks, panel III: Cryo-EM images of micelles formed at 0.012 mM in the presence of 0.106 mM OA, panels IV–V: confocal 3D projections of OA droplets in 0.012 mM PS80 with 0.106 mM OA obtained from (IV) summed Z-projections of the CLSM Z stacks and (V) a series of individual Z-stack slices, (c) dynamic surface tension for 0.012 mM PS80 with different concentrations of OA (0.017, 0.035, 0.071, and 0.106 mM), (d) normalized reflectivity (*R*/*R*_f_) of XRR measurements from adsorbed layers OA/PS80 at the air–water interface as a function of *Q*_*z*_ at 0.012 mM of PS80 and different bulk concentrations of OA (0.017, 0.035, 0.071, and 0.106 mM), the upper four XRR curves are shifted by 0.5 for clarity, (e) EDP as a function of the distance *z* normal to the surface at 0.012 mM of PS80 and different bulk concentrations of OA (0.017, 0.035, 0.071, and 0.106 mM).

Cryo-EM and confocal microscopy were employed as complementary methods to support and extend the DLS characterization of PS80 micelles and OA droplets (Figure 3b). Cryo-EM was chosen to resolve the nanoscale structure of micelles, whereas confocal microscopy provided 3D visualization of larger OA droplets within the same samples, both techniques allowing correlation with DLS results. Two samples were prepared for visualization: 0.012 mM PS80 with and without 0.106 mM OA. Panels I (only PS80) and IV (PS80 + OA) show Cryo-EM images. In panel I, spherical PS80 micelles with an average diameter of ∼11 nm are observed, consistent with DLS measurements (Figure S1). While 0.012 mM is below the nominal cmc of PS80, a relatively small number of micelles exist due to gradual micellization,^61^ which can give rise to small spherical micelle-like assemblies (indicated by the arrow in the Figure 3b-panel I) below the equilibrium cmc. Panel III reveals an increase in micelle size upon OA addition, indicating that OA drives additional PS80 monomers into micelles (indicated by the arrow), consistent with the DLS results (Figures 3a and S2). To visualize OA droplets, the same samples were imaged by confocal microscopy to correlate with the larger DLS peak. Confocal 3D projections of 0.012 mM PS80 alone serve as a baseline. Panel II shows summed Z-projections of CLSM Z-stacks. As expected, no fluorescence signal is detected in the absence of OA. Confocal 3D projections of the OA-containing sample (panels IV–V) reveal distinct droplets. Once micelles become saturated, excess insoluble OA forms spherical droplets, visualized in panel IV (summed Z-projection). The presence of droplets observed by confocal microscopy is consistent with the secondary ∼300 nm population detected by DLS (Figure 3a), although confocal imaging is used here only qualitatively.

Figure 3c displays the surface tension relaxation curves for PS80/OA solutions. The curves reveal a decreased equilibrium surface tension with increasing OA concentrations. As the OA concentration increases in the bulk solution, the relaxation times are also faster. OA, being an insoluble surfactant, is highly surface-active and accelerates the reduction in surface tension. This behavior suggests that the mixed monolayers of OA and PS80 are a result of adsorption from the bulk phase, and the presence of OA in the monolayer results in a reduction in tension.

While dynamic surface tension measurements can show that oleic acid molecules from micelles are adsorbing to the air–water interface, these measurements alone cannot provide the detailed structural and compositional information on the interfacial layer. Therefore, X-ray reflectivity measurements are conducted to precisely quantify the behavior of the mixed solutions at the air–water interface. These measurements were performed for solutions with a fixed PS80 concentration of 0.012 mM and varying OA concentrations of 0.017, 0.035, 0.071, and 0.106 mM. The specular reflection data, shown in Figure 3d, demonstrates a narrowing of the reflectivity curves with increasing OA concentration, suggesting the surface becomes saturated with a mixed layer of OA and PS80.

The electron density profile in Figure 3e, derived from the fitted reflectivity data further supports these observations. The profile reveals that as the OA concentration in the bulk solution increases both the electron density and the thickness of the interfacial layer (Table S3). Upon addition of OA to the solution, our XRR fits (thickness and electron density parameters; Tables S3–S5, Supporting Information) indicate that the first fitted layer at the air–water boundary is dominated by the hydrocarbon chains of OA, with a minor contribution from PS80 tails due to interfacial packing. The second layer corresponds to a hydrated region comprising the OA carboxylic headgroups interspersed with part of PS80s ethoxylate chains, while the third layer represents the remaining portion of the PS80 headgroup extending further into the aqueous phase. These results suggest that OA, with its relatively small headgroup, preferentially resides in the aqueous-side of the interfacial layer but close to the air, while its hydrocarbon tail occupies the region adjacent to the vapor phase.

Surface concentrations of PS80 and OA were calculated using the same method described above (see Supporting Information). Table 4 presents the surface concentrations of PS80 and OA at the air–water interface for varying OA concentrations. The results show that the concentration of OA at the interface increases from 1.12 mg/m^2^ to 1.36 mg/m^2^ as the bulk OA concentration rises. This suggests that higher OA concentrations lead to more OA molecules adsorbing at the surface, thereby altering the composition of the interfacial layer as PS80 and OA coadsorb.

**Table 4:** Summary of X-ray Reflectivity Measurements of Surface Concentrations for Constant Bulk Concentration of Polysorbate 80 at 0.012 mM and Different Bulk Concentration of Oleic Acid^a^

$\frac{OA}{PS80}\left(bulk\right)$	$\frac{OA}{PS80}\left(surface\right)$	$\Gamma_{PS80}\left(\frac{mg}{m^{2}}\right)$	$\Gamma_{OA}\left(\frac{mg}{m^{2}}\right)$	$\Gamma_{W}\left(\frac{mg}{m^{2}}\right)$
0	0	1.71 ± 0.63	0	1.72 ± 0.57
1.42	4.30 ± 2.16	1.20 ± 0.35	1.12 ± 0.46	1.54 ± 0.37
2.92	5.68 ± 1.87	1.03 ± 0.23	1.27 ± 0.31	1.43 ± 0.25
5.92	5.69 ± 1.35	1.09 ± 0.18	1.34 ± 0.23	1.51 ± 0.19
8.84	5.76 ± 2.71	1.10 ± 0.35	1.36 ± 0.47	1.52 ± 0.38

a Parameters: $\frac{OA}{PS80}$ is the molar ratio of OA and PS80, Γ_PS80_ is the surface concentration of PS80, Γ_OA_ is the surface concentration of oleic acid, Γ_W_ is the surface concentration of water, uncertainties reflect standard error propagation from MCMC-derived XRR fitted parameters.

To better understand the saturation behavior, we examined the molar ratio of OA to PS80 in both the bulk and at the interface. As shown in Table 4, at a PS80 concentration of 0.012 mM, the OA/PS80 ratio in the bulk increases progressively. Once the bulk OA/PS80 ratio exceeds ∼3, the corresponding surface ratio of OA/PS80 plateaus to ∼5.7, suggesting saturation of the interface. This implies that additional OA beyond this point remains in the bulk, contributing to droplet formation, rather than further enriching the surface layer.

Figure 4 illustrates the competitive adsorption of PS80 and OA at higher bulk concentrations of PS80 (0.06 mM and 0.12 mM) with varying OA concentrations (0.017, 0.035, 0.071, and 0.106 mM). Figure 4a shows the volume percentage as a function of particle size, indicating that OA molecules are solubilized within the micelles of PS80, forming a homogeneous mixture. For all OA concentrations at both 0.06 mM and 0.12 mM PS80, a single DLS peak is observed around 11 nm, representing the size of the micelles. At these higher concentrations of PS80, the population of micelles is sufficient to accommodate all the OA, resulting in a single peak, in contrast to the multiple peaks seen at 0.012 mM of PS80, where OA could not be fully solubilized into the micelles. In addition, no measurable shift in micelle size is observed upon OA addition at higher PS80 concentrations, which is attributed to the ensemble-averaged nature of DLS measurements, where small incremental changes in hydrodynamic diameter fall below the technique’s resolution.

**Figure 4:**
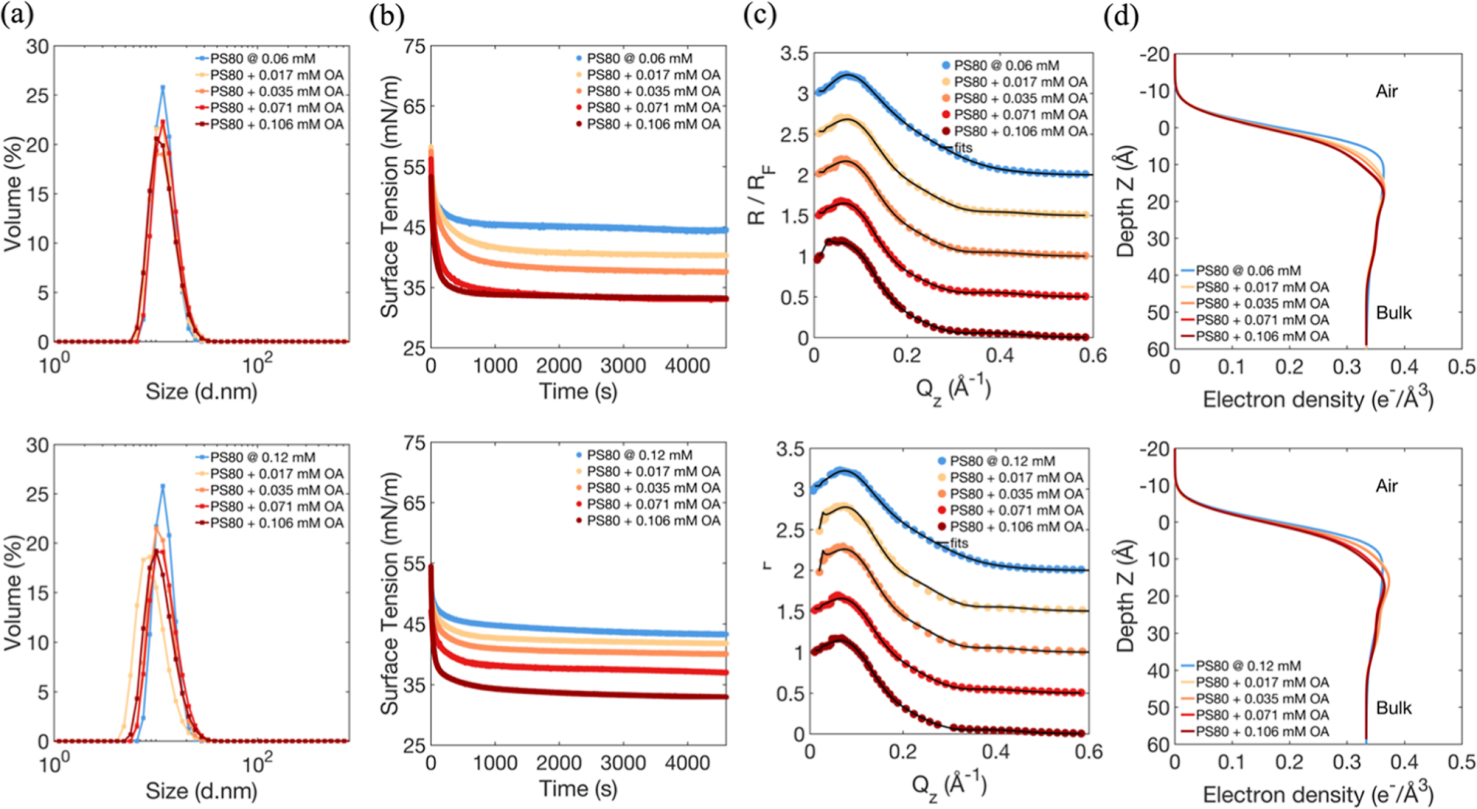
(a) Dynamic light scattering volume distribution as a function of size for solutions of 0.06 mM, and 0.12 mM of PS80 with different concentrations of OA, (b) dynamic surface tension for solutions of 0.06 mM, and 0.12 mM of PS80 with different concentrations of OA (0.017, 0.035, 0.071, and 0.106 mM), (c) normalized reflectivity (*R*/*R*_f_) of XRR measurements from adsorbed layers OA/PS80 at the air–water interface as a function of *Q*_*z*_ normal to the surface at 0.06 mM and 0.12 mM of PS80 and different bulk concentrations of OA, the upper four XRR curves are shifted by 0.5 for clarity, (d) EDP as a function of the distance *z* normal to the surface of at 0.06 mM and 0.12 mM of PS80 and different bulk concentrations of OA.

Figure 4b presents the relaxation time data for solutions with higher PS80 concentrations (0.06 mM and 0.12 mM) and varying OA concentrations. As observed in the 0.012 mM of PS80 system, increasing OA concentration leads to a significant decrease in surface tension. However, for higher bulk PS80 concentrations, the quasi-equilibrium surface tension shifts to slightly higher values relative to the 0.012 mM (Figure 3c). This shift can be attributed to the increased dilution of OA molecules into a greater number of micelles, reducing the concentration of OA transported to the interface per micelle. This decrease in the ratio of OA-to-PS80 at the interface results in a smaller change in the surface tension.

To gain a deeper understanding of the composition of the interfacial layer, X-ray reflectivity measurements were conducted for 0.06 mM and 0.12 mM PS80 solutions with varying OA concentrations (0.017 to 0.106 mM). Figure 4c presents the reflectivity data, showing a distinct trend of narrowing reflectivity curves as the OA concentration increases. This shift to lower *Q*_*z*_ values suggests that OA is adsorbing to the surface together with PS80, further altering the interfacial structure.

The electron density profiles, shown in Figure 4d, were derived by fitting a slab model to the reflectivity data. These profiles were used to calculate the surface concentration of both components (Supporting Information). Figure 5 and Tables S6 and S7 summarize the surface concentration calculations for 0.06 mM and 0.12 mM PS80, respectively. The results are consistent with trends observed in surface tension measurements. The results show that as the OA concentration in the bulk solution increases, more OA molecules are adsorbed to the surface (Figure 5). However, compared to the 0.012 mM system, the surface concentrations of OA are lower in both 0.06 and 0.12 mM PS80 solutions. For instance, at a bulk concentration of 0.071 mM OA, the surface concentrations of OA are 1.34, 1.04, and 0.84 mg/m^2^ for 0.012, 0.06, and 0.12 mM PS80, respectively. These findings indicate that at higher PS80 concentrations in the bulk, the OA molecules are more diluted in micellar assemblies, leading to a reduced surface concentration at the air–water interface.

**Figure 5:**
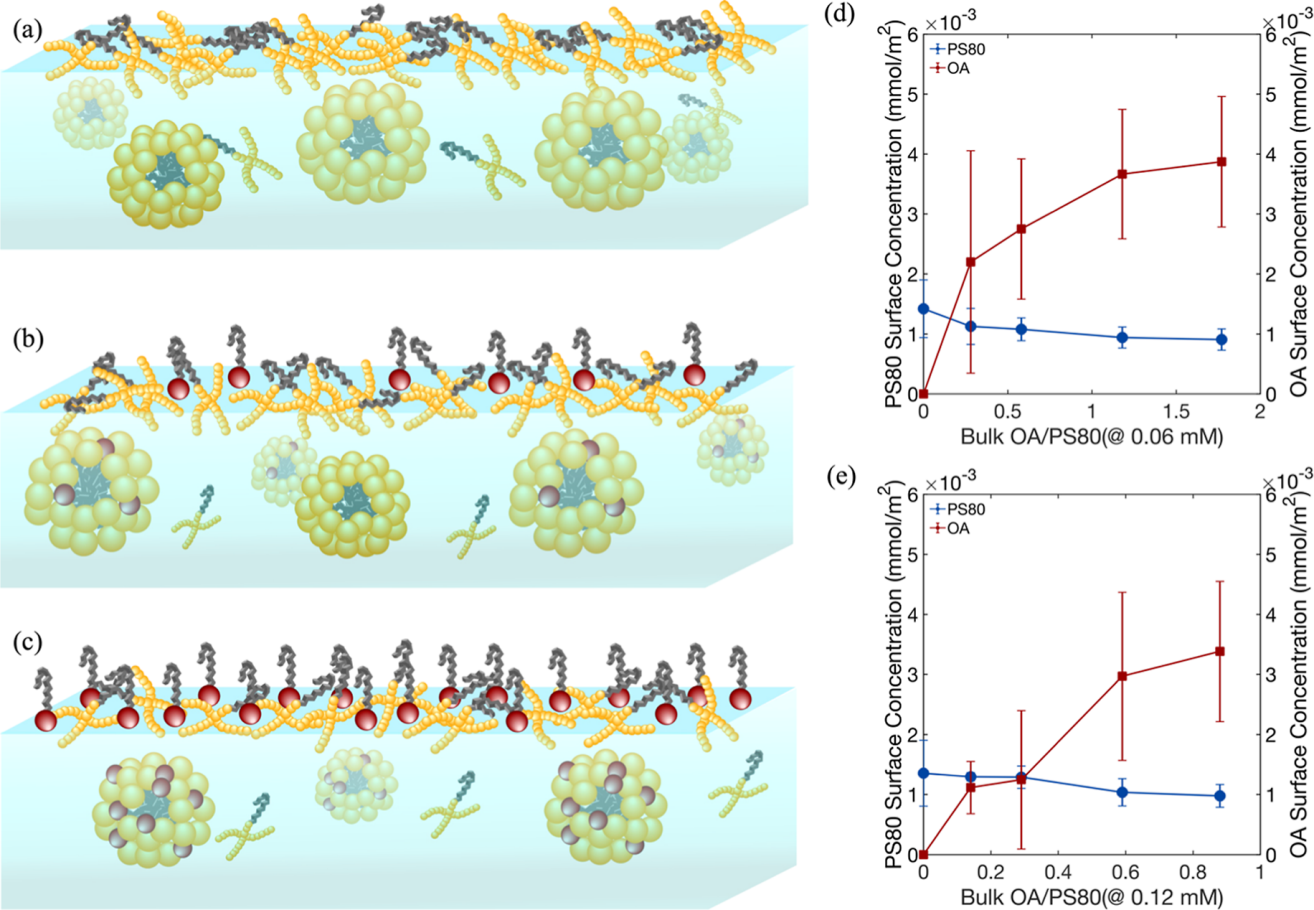
Schematic representation of PS80 and OA adsorption at the air–water interface: (a) single-component system, PS80 adsorbs to the interface, (b) introduction of OA to the solution, coadsorption of PS80 and OA begins, with OA transferring to the interface via micelles, (c) at higher OA concentrations, increased adsorption occurs, (d) PS80 and OA surface concentrations as a function of bulk OA/PS80 concentration ratio for PS80 at concentration of 0.06 mM, (e) PS80 and OA surface concentrations as a function of bulk OA/PS80 concentration ratio for PS80 at concentration of 0.12 mM, error bars represent uncertainty reflect standard error propagation from MCMC-derived XRR fitted parameters.

This dilution effect is also reflected in the OA to PS80 molar ratios in both the bulk and at the surface (Tables S6 and S7). At higher PS80 concentrations (0.06 and 0.12 mM), the surface OA-to-PS80 ratios remain well below the threshold associated with OA droplet formation observed at 0.012 mM. This suggests that although OA continues to dominate the interfacial composition, its accumulation is reduced due to enhanced solubilization within PS80 micelles, maintaining the system below the threshold for OA droplet formation observed at lower PS80 concentrations.

## Conclusion

This study establishes that oleic acid (OA) is solubilized within PS80 micelles, which act as the dominant carriers transporting OA to the air–water interface. At the interface, the adsorption of mixed micelles defines surface composition, as confirmed by DLS, surface tension, and X-ray reflectivity measurements. Together, these techniques reveal that mixed OA/PS80 layers directly shape interfacial properties, producing lower surface tension and higher electron density with increasing OA concentration.

We also found that this relationship is not linear; at higher PS80 concentrations, the adsorption of OA at the surface is reduced. This behavior is likely due to the increased number of micelles in the bulk solution, which dilutes the OA and limits its ability to accumulate at the interface. Moreover, when OA saturates micelles and droplet formation occurs, further increases in bulk OA result in only minor shifts in the interfacial composition, indicating approach toward saturation of the mixed layer at the air–water interface. This finding underscores the complex interplay between bulk-phase composition and interfacial behavior, highlighting the crucial role of PS80 in regulating OA’s adsorption and demonstrating that increasing the overall concentration of surfactant can, counterintuitively, inhibit the accumulation of hydrophobic species at the interface.

In conclusion, our work provides a quantitative framework for understanding the interactions between surfactants and hydrophobic amphiphiles at the air–water interface. By directly linking bulk concentration to interfacial composition and properties, this research offers critical insights for designing more robust pharmaceutical and food formulations where the stability of protein-based therapeutics and other colloidal systems relies on precise control over interfacial phenomena. The methodology employed here, particularly the use of X-ray reflectivity, serves as a powerful tool for resolving molecular-level details of multicomponent systems adsorbing to air–water interfaces.

## Supplementary Material



## Notes

Langmuir adsorption fit for PS80; X-ray reflectivity data analysis; surface concentration calculation of oleic acid monolayer spread on pure water; surface concentration calculation for single component system (PS80); surface concentration calculation for mixed component system (PS80 + OA); fitting parameters from XRR measurements for PS80, fitting parameters from XRR measurements for different concentrations of mixed system (PS80 + OA); summary of XRR surface concentrations for mixed system (PS80 + OA); dynamic light scattering data (PDF)

## References

[1] Tanford, C. Hydrophobic Effect: Formation of Micelles and Biological Membranes; Wiley, 1980.

[2] Israelachvili, J. N. Intermolecular and Surface Forces; Academic Press, 2010.

[3] Nagarajan R. (2002). Molecular
Packing Parameter and Surfactant Self-Assembly: The Neglected Role
of the Surfactant Tail. Langmuir.

[4] Douliez J.-P., Houssou B. H., Fameau A.-L., Navailles L., Nallet F., Grélard A., Dufourc E. J., Gaillard C. (2016). Self-Assembly
of Bilayer Vesicles Made of Saturated Long Chain Fatty Acids. Langmuir.

[5] Dukhin, S. S. ; Kretzschmar, G. ; Miller, R. Dynamics of Adsorption at Liquid Interfaces: Theory, Experiment, Application; Elsevier, 1995.

[6] Nagarajan R. (1996). Solubilization
in Aqueous Solutions of Amphiphiles. Curr. Opin.
Colloid Interface Sci..

[7] Macierzanka A., Torcello-Gómez A., Jungnickel C., Maldonado-Valderrama J. (2019). Bile Salts in Digestion and Transport of Lipids. Adv. Colloid Interface Sci..

[8] Naso J. N., Bellesi F. A., Pizones
Ruiz-Henestrosa V. M., Pilosof A. M. R. (2021). A New
Methodology to Assess the Solubility of Fatty Acids: Impact of Food
Emulsifiers. Food Res. Int..

[9] Naso J. N., Bellesi F. A., Pizones Ruiz-Henestrosa V. M., Pilosof A. M. R. (2022). Solubilization
of Lipolysis Products in Mixed Micelles Is Enhanced in Presence of
Bile Salts and Tween 80 as Revealed by a Model Study (Oleic Acid)
and Emulsified Chia-Oil. Food Res. Int..

[10] Tanojo H., Junginger H. E., Boddé H. E. (1997). In Vivo
Human Skin Permeability Enhancement
by Oleic Acid: Transepidermal Water Loss and Fourier-Transform Infrared
Spectroscopy Studies. J. Controlled Release.

[11] Boelsma E., Tanojot H., Boddét H. E., Ponec M. (1996). Assessment of the Potential
Irritancy of Oleic Acid on Ruman Skin: Evaluation In Vitro and In
Vivo. Toxicol. In Vitro.

[12] Johansson I., Svensson M. (2001). Surfactants Based on Fatty Acids and Other Natural
Hydrophobes. Curr. Opin. Colloid Interface Sci..

[13] Kováčik A., Kopečná M., Hrdinová I., Opálka L., Boncheva Bettex M., Vávrová K. (2023). Time-Dependent
Differences in the Effects of Oleic Acid and Oleyl Alcohol on the
Human Skin Barrier. Mol. Pharmaceutics.

[14] Lee Y.-C., Dalton C., Regler B., Harris D. (2018). Drug Solubility in
Fatty Acids as a Formulation Design Approach for Lipid-Based Formulations:
A Technical Note. Drug Dev. Ind. Pharm..

[15] Kalhapure R. S., Akamanchi K. G. (2012). Oleic Acid
Based Heterolipid Synthesis, Characterization
and Application in Self-Microemulsifying Drug Delivery System. Int. J. Pharm..

[16] Rangel-Yagui C. O., Pessoa A., Tavares L. C. (2005). Micellar Solubilization
of Drugs. J. Pharm. Pharm. Sci..

[17] Torchilin V. P. (2006). Micellar
Nanocarriers: Pharmaceutical Perspectives. Pharm.
Res..

[18] La
Sorella G., Strukul G., Scarso A. (2015). Recent Advances in
Catalysis in Micellar Media. Green Chem..

[19] Subraveti S. N., Nader M. G., AziziHariri P., John V. T., Lamichhane N., Raghavan S. R. (2024). Vesicle–Micelle
Transitions Driven by ROS, Light
and Heat. Nanoscale.

[20] Ghezzi M., Pescina S., Delledonne A., Ferraboschi I., Sissa C., Terenziani F., Remiro P. D. F. R., Santi P., Nicoli S. (2021). Improvement of Imiquimod Solubilization
and Skin Retention via TPGS Micelles: Exploiting the Co-Solubilizing
Effect of Oleic Acid. Pharmaceutics.

[21] Kalhapure R. S., Akamanchi K. G. (2013). A Novel Biocompatible Bicephalous
Dianionic Surfactant
from Oleic Acid for Solid Lipid Nanoparticles. Colloids Surf., B.

[22] Sobhani H., Tarighi P., Ostad S. N., Shafaati A., Nafissi-Varcheh N., Aboofazeli R. (2018). Rapamycin-Loaded,
CapryolTM 90 and Oleic Acid Mediated
Nanoemulsions: Formulation Development, Characterization and Toxicity
Assessment. Iran. J. Pharm. Res..

[23] Golding M., Wooster T. J. (2010). The Influence of Emulsion Structure and Stability on
Lipid Digestion. Curr. Opin. Colloid Interface
Sci..

[24] Tzocheva S. S., Kralchevsky P. A., Danov K. D., Georgieva G. S., Post A. J., Ananthapadmanabhan K.
P. (2012). Solubility Limits and
Phase Diagrams for Fatty Acids in Anionic (SLES) and Zwitterionic
(CAPB) Micellar Surfactant Solutions. J. Colloid
Interface Sci..

[25] Mitrinova Z., Tcholakova S., Popova Z., Denkov N., Dasgupta B. R., Ananthapadmanabhan K.
P. (2013). Efficient Control of
the Rheological
and Surface Properties of Surfactant Solutions Containing C8–C18
Fatty Acids as Cosurfactants. Langmuir.

[26] Xu W., Nikolov A., Wasan D. T., Gonsalves A., Borwankar R. P. (2003). Foam Film Rheology and Thickness
Stability of Foam-Based
Food Products. Colloids Surf., A.

[27] Khan T. A., Mahler H.-C., Kishore R. S. K. (2015). Key
Interactions of Surfactants in
Therapeutic Protein Formulations: A Review. Eur. J. Pharm. Biopharm..

[28] Wu M.-H., Yan H. H., Chen Z.-Q., He M. (2017). Effects of Emulsifier
Type and Environmental Stress on the Stability of Curcumin Emulsion. J. Dispers. Sci. Technol..

[29] Kralova I., Sjöblom J. (2009). Surfactants Used in Food Industry:
A Review. J. Dispers. Sci. Technol..

[30] Kanthe A. D., Krause M., Zheng S., Ilott A., Li J., Bu W., Bera M. K., Lin B., Maldarelli C., Tu R. S. (2020). Armoring the Interface with Surfactants
to Prevent the Adsorption
of Monoclonal Antibodies. ACS Appl. Mater. Interfaces.

[31] Kishore R. S. K., Pappenberger A., Dauphin I. B., Ross A., Buergi B., Staempfli A., Mahler H.-C. (2011). Degradation of Polysorbates 20 and
80: Studies on Thermal Autoxidation and Hydrolysis. J. Pharm. Sci..

[32] Weber J., Buske J., Mäder K., Garidel P., Diederichs T. (2023). Oxidation
of Polysorbates – An Underestimated Degradation Pathway?. Int. J. Pharm. X.

[33] Allmendinger A., Lebouc V., Bonati L., Woehr A., Kishore R. S. K., Abstiens K. (2021). Glass Leachables as
a Nucleation Factor for Free Fatty
Acid Particle Formation in Biopharmaceutical Formulations. J. Pharm. Sci..

[34] Kanthe A. D., Carnovale M. R., Katz J. S., Jordan S., Krause M. E., Zheng S., Ilott A., Ying W., Bu W., Bera M. K., Lin B., Maldarelli C., Tu R. S. (2022). Differential Surface Adsorption Phenomena for Conventional and Novel
Surfactants Correlates with Changes in Interfacial mAb Stabilization. Mol. Pharmaceutics.

[35] Kishore R. S. K., Kiese S., Fischer S., Pappenberger A., Grauschopf U., Mahler H.-C. (2011). The Degradation
of Polysorbates 20
and 80 and Its Potential Impact on the Stability of Biotherapeutics. Pharm. Res..

[36] Gong Y., Yang W., Wu C., Fan X., Zhang X., Li J., Wu D. (2024). Design of Tween80/Oleic
Acid Composite Vesicle and
Its Application in Controlled Release of Vitamin C. Colloid Polym. Sci..

[37] Zürcher D., Wuchner K., Arosio P. (2024). Mitigation Strategies
against Antibody
Aggregation Induced by Oleic Acid in Liquid Formulations. Mol. Pharmaceutics.

[38] Siska C. C., Pierini C. J., Lau H. R., Latypov R. F., Matthew
Fesinmeyer R., Litowsk J. R. (2015). Free Fatty Acid Particles in Protein
Formulations, Part 2: Contribution of Polysorbate Raw Material. J. Pharm. Sci..

[39] Larson N. R., Wei Y., Prajapati I., Chakraborty A., Peters B., Kalonia C., Hudak S., Choudhary S., Esfandiary R., Dhar P., Schöneich C., Middaugh C. R. (2020). Comparison of Polysorbate
80 Hydrolysis and Oxidation on the Aggregation of a Monoclonal Antibody. J. Pharm. Sci..

[40] NSF’s ChemMatCARS, Sector 15 at Advanced Photon Source. XModFit, Https://Chemmatcars.Github.Io/XModFit/ (accessed Nov 21, 2025).

[41] Richter A.
G., Kuzmenko I. (2013). Using in Situ
X-Ray Reflectivity to Study Protein Adsorption
on Hydrophilic and Hydrophobic Surfaces: Benefits and Limitations. Langmuir.

[42] Tolan, M. X-Ray scattering from Soft-Matter Thin Films, Springer Tracts in Modern Physics; Springer, 1988.

[43] Zhang P., Pham T., Liu C., Leon Plata P., Kalkowski J., Cheng G., Bu W., Lin B., Liu Y. (2020). Impeded Molecular Reorganization by Polyethylene Glycol Conjugation
Revealed by X-Ray Reflectivity and Diffraction Measurements. Langmuir.

[44] Sallaberry C. A., Voss B. J., Stone W. B., Estrada F., Bhatia A., Soto J. D., Griffin C. W., Vander
Zanden C. M. (2023). Curcumin
Reduces Amyloid Beta Oligomer Interactions with Anionic Membranes. ACS Chem. Neurosci..

[45] Voss L. F., Bazerbashi M. F., Beekman C. P., Hadad C. M., Allen H. C. (2007). Oxidation
of Oleic Acid at Air/Liquid Interfaces. J. Geophys.
Res.:Atmos..

[46] Gonçalves
Da Silva A. M., Romão R. I. S. (2005). Mixed Monolayers Involving DPPC,
DODAB and Oleic Acid and Their Interaction with Nicotinic Acid at
the Air–Water Interface. Chem. Phys.
Lipids.

[47] Vaknin D., Bu W. (2010). Neutrally Charged Gas/Liquid
Interface by a Catanionic Langmuir Monolayer. J. Phys. Chem. Lett..

[48] Torrent-Burgués J. (2018). Thermodynamic
Behaviour of Mixed Films of an Unsaturated and a Saturated Polar Lipid.
(Oleic Acid-Stearic Acid and POPC-DPPC). Colloids
Interfaces.

[49] Gaines, G. L. Insoluble Monolayers at Liquid–Gas Interfaces; Interscience Publisher, 1966.

[50] Hwan
Ha T., Kyu Kim D., Choi M.-U., Kim K. (2000). Influence of Poly­(Ethylenimine)
on the Monolayer of Oleic Acid at the Air/Water Interface. J. Colloid Interface Sci..

[51] Horiuchi S., Winter G. (2015). CMC Determination of Nonionic Surfactants in Protein
Formulations Using Ultrasonic Resonance Technology. Eur. J. Pharm. Biopharm..

[52] Patist A., Bhagwat S. S., Penfield K. W., Aikens P., Shah D. O. (2000). On the
Measurement of Critical Micelle Concentrations of Pure and Technical-grade
Nonionic Surfactants. J. Surfactants Deterg..

[53] Strickley R. G., Lambert W. J. (2021). A Review of Formulations
of Commercially Available
Antibodies. J. Pharm. Sci..

[54] Kerwin B. A. (2008). Polysorbates
20 and 80 Used in the Formulation of Protein Biotherapeutics: Structure
and Degradation Pathways. J. Pharm. Sci..

[55] Glenn K. M., Moroze S., Bhattacharya S. C., Palepu R. M. (2005). Effect of Ethylene
Glycol on the Thermodynamic and Micellar Properties of Tween 40, 60,
and 80. J. Dispers. Sci. Technol..

[56] Tang X., Huston K. J., Larson R. G. (2014). Molecular
Dynamics Simulations of
Structure–Property Relationships of Tween 80 Surfactants in
Water and at Interfaces. J. Phys. Chem. B.

[57] Luz A. M., Barbosa G., Manske C., Tavares F. W. (2023). Tween-80 on Water/Oil
Interface: Structure and Interfacial Tension by Molecular Dynamics
Simulations. Langmuir.

[58] Huston K. J., Larson R. G. (2015). Reversible and Irreversible
Adsorption Energetics of
Poly (Ethylene Glycol) and Sorbitan Poly (Ethoxylate) at a Water/Alkane
Interface. Langmuir.

[59] King T. E., Humphrey J. R., Laughton C. A., Thomas N. R., Hirst J. D. (2024). Optimizing
Excipient Properties to Prevent Aggregation in Biopharmaceutical Formulations. J. Chem. Inf. Model..

[60] Deechongkit S., Wen J., Narhi L. O., Jiang Y., Park S. S., Kim J., Kerwin B. A. (2009). Physical and Biophysical
Effects of Polysorbate 20
and 80 on Darbepoetin Alfa. J. Pharm. Sci..

[61] Knoch H., Ulbrich M. H., Mittag J. J., Buske J., Garidel P., Heerklotz H. (2021). Complex Micellization
Behavior of the Polysorbates
Tween 20 and Tween 80. Mol. Pharmaceutics.

