# Supplementary material for: Adsorption of Mixed Micelles of Polysorbate 80 and Oleic Acid to the Air–Water Interface
Source: Langmuir. 2026 Jan 5;42(2):2200–11. doi: 10.1021/acs.langmuir.5c05532 (PMC12825370; doi:10.1021/acs.langmuir.5c05532)
Supplement: Supplementary file 1 [file la5c05532_si_001.pdf]

Supporting Information

Adsorption of mixed micelles of polysorbate 80 and oleic acid to the air-water interface

Nooshin Sadat Ayati[a], Ankit D. Kanthe[b], Mary Krause[b], Songyan Zheng[b], Honghu Zhang[c], Luis E. Ortuno Macias[a], Charles Maldarelli[a], Raymond S. Tu*[a]*

a Department of Chemical Engineering, City College of New York, New York, NY, 10031, US

b Drug Product Development, Bristol Myers Squibb, New Brunswick, NJ, 08901, US

* Corresponding authors: Department of Chemical Engineering, City College of New York, New York, NY, 10031, US

Email: tu@ccny.cuny.edu (Raymond S. Tu)

Email: cmaldarelli@ccny.cuny.edu (Charles Maldarelli)

C National Synchrotron Light Source II, Brookhaven National Laboratory, Upton, NY, 11973, USA

Langmuir adsorption fit

To estimate the maximum surface coverage (Γ_∞), the equilibrium surface tension (γ) as a function of bulk concentration (C_s) is fitted to the Gibbs equation within the Langmuir adsorption framework. The equilibrium surface tension of PS80 alone, over the concentration range of 1.2 × 10^{-3} to 2.4 × 10^{-2} mM, is plotted as a function of the bulk concentration of PS80 on a semi-logarithmic scale, along with the corresponding Langmuir fitting parameters[1,2]. The adsorption isotherms and equation of state are provided by:

$$\gamma = \gamma_c + RT\Gamma_\infty \, ln\left(\frac{1}{1+\frac{C_s}{a}}\right) \quad S(1)$$

Where Γ_∞ is the estimate of the excess surface concentration and *a* is the ratio of the desorption to adsorption rate constants. We note that the fit using Langmuir isotherm is good giving Γ_∞=2.54 mg/m^2 (1.94*10^{-6} mol/m^2) and a=1.058*10^{-7} mol/L.

X-ray reflectivity data analysis

The X-ray reflectivity data are normalized by the Fresnel reflectivity (R_f) of water, which represents the reflectivity from an ideally smooth surface. The ratio (R/R_f) as a function of the

wave vector transfer perpendicular to the surface (Q_z) is fitted to slab models to obtain the electron density distribution across the interfacial layer. Both two- and three-slab models are used to fit the reflectivity data, providing insights into the molecular features at the surface, such as electron density, thickness, and interfacial roughness. These parameters are then used to calculate the surface concentrations of PS80, and OA adsorbed at the air-water interface from the bulk solution.

The molecular weight, molecular volume, and number of electrons for PS80 and OA are reported in Table S1. For the PS80 system, the molecular volume was calculated based on the head and tail group volumes, as described in the literature[3–5]. The tail group volume is estimated to be half the molecular volume of the DOPC tail group (492 Å^3 per PS80 tail), and the total head group volume is 1311 Å^3. Therefore, the total molecular volume of PS80 is 1803 Å^3. For OA, the molecular volume is estimated as 492 Å^3 for the tail group (similar to the PS80 tail group) and 25.8 Å^3 for the carboxyl head group[6], yielding a total molecular volume of 517.8 Å^3.

Table S1. The molecular weight (M_W), number of electrons (e) and molecular volume (v) for the PS80, OA and water used in the analysis of XRR data.

Component	MW (g/mol)	e	ν (Å^3)
**PS80**	1310	716	1803
**OA**	282.46	158	517.8
**Water**	18	10	30

To calculate the surface concentrations of PS80 molecules at the interface, a two-slab model is used to fit the reflectivity curve. The corresponding fitting parameters for PS80 molecules are provided in Table S2. For PS80 molecules, due to the larger head group compared to OA, the slabs are not separated. Instead, the total thickness and electron density of the model are used for the calculation of the surface concentration.

For the mixed-component system (PS80 + OA), three-slab models are used to fit the reflectivity curves. The fitting parameters for the PS80 + OA system at different bulk concentrations of PS80 (0.012, 0.06, and 0.12 mM) and varying OA concentrations (0.017, 0.035, 0.071 and 0.106 mM) are presented in Tables S3, S4, and S5, respectively.

Oleic acid monolayer spread on pure water

The surface concentration of oleic acid (OA) molecules in the monolayer spread on the water substrate is calculated using a molar volume balance and total electron density balance for each slab constituting the air-water interface. The concentration of OA at the air-water interface, denoted as Γ_s (molecule/Å^2), is determined by the following equation:

$$d_1 \times \rho_1 = \Gamma_s \times e_s^T \qquad (S2)$$

Where d_1 (Å) and ρ_1 (e/Å^3) as the thickness and the electron density of first slab (tail group) respectively, and e_s^T (e/molecule) as the number of electrons contained in the oleic acid tail group

(135 e/molecule). To compute the concentration of water molecules in that region we used fitting parameters of second slab (head group) using the following equation.

$$d_2 \times \rho_2 = \Gamma_s \times e_s^H + \Gamma_w \times e_w \qquad (S3)$$

Where Γ_s is obtained from the first slab, d_2 (Å) and ρ_2 (e/Å^3) are the thickness and the electron density of head group respectively, and Γ_w (molecule/Å^2) is the surface concentration of water molecules, e_s^H (e/molecule) is the number of electrons in the polar head group of OA (23 e/molecule), and e_w (e/molecule) the number of electrons in each water molecule (10 e/molecule).

Surface concentration for single component system (PS80)

Similar to the OA surface concentration calculation, we applied electron density and molar volume balances. However, we used the total electron density (e_T) and total thickness (d_T) for the calculation of PS80 resulting in:

$$d_T = \left(\Gamma_{ps80} \times \nu_{ps80}\right) + \left(\Gamma_w \times \nu_w\right) \qquad S(4)$$

$$e_T = \left(\Gamma_{ps80} \times e_{ps80}\right) + \left(\Gamma_w \times e_w\right) \qquad S(5)$$

Where d_T (Å) is the total thickness of the layers, e_T (e/Å^2) is the total number of electron of the air-water interface, e_{PS80} is the number of electrons in PS80 molecules (716 e/molecule), e_w is the number of electrons in each water molecules (10 e/molecule), Γ_{PS80} (molecule/Å^2) is the surface

concentration of PS80, Γ_w (molecule/Å^2) is the surface concentration of water molecules, ν_{PS80} is the molecular volume of the PS80 (Å^3), ν_w is the molecular volume of water (Å^3).

Surface concentration for mixed component system (PS80+OA)

For a two-component system, the electron density and molar volume balances, similar to those used in a single-component system, provide a framework to calculate the surface concentrations of PS80 and oleic acid (OA). Where is the total thickness of the layers d_T (Å) and the total electron density e_T (e/Å^2) at the air/water interface are given by the following equations:

$$d_T = \left(\Gamma_{ps80} \times \nu_{ps80}\right) + \left(\Gamma_w \times \nu_w\right) + \left(\Gamma_{OA} \times \nu_{OA}\right) \qquad S(6)$$

$$e_T = \left(\Gamma_{ps80} \times e_{ps80}\right) + \left(\Gamma_w \times e_w\right) + \left(\Gamma_{OA} \times e_{OA}\right) \qquad S(7)$$

Where e_{PS80} is the number of electrons in PS80 molecules (716 e/molecule), e_w is the number of electrons in each water molecules (10 e/molecule), e_{OA} is the number of electrons in OA molecules (158 e/molecule), Γ_{PS80} (molecule/Å^2) is the surface concentration of PS80, Γ_{OA} (molecule/Å^2) is the surface concentration of oleic acid at air-water interface, Γ_w (molecule/Å^2) is the surface concentration of water molecules, ν_{PS80} is the molecular volume of the PS80 (Å^3), ν_{OA} is the molecular volume of the OA (Å^3), ν_w is the molecular volume of water (Å^3).

To quantitatively predict the surface concentration of each component during co-adsorption, we assume that the surface structure of each component (PS80 or OA) remains unchanged from their respective single-component systems. Under this assumption, the surface concentration of water

molecules (Γ_w) is calculated based on the number of water molecules associated with the head group of OA and PS80, with the number of water molecules remaining constant from the single-component system for each material. Once the surface concentration of water molecules is determined, the surface concentrations of OA and PS80 can be calculated using the equations outlined above. This approach provides a reliable estimation of the interfacial composition during mixed-component adsorption.

Table S2. Fitting parameters from XRR measurements from adsorbed layers of polysorbate 80 at different bulk concentration.

0.0012 mM

Slab #	**d** (Å)		**ρ** ($\frac{e}{Å^3}$)		**σ** (Å)	
1	6.09	± 0.045	0.4090	± 0.00106	3.31	± 0.0093
2	17.84	± 0.074	0.3459	± 0.00053	9.85	± 0.0761

0.012 mM

Slab #	**d** (Å)		**ρ** ($\frac{e}{Å^3}$)		**σ** (Å)	
1	22.22	± 0.43	0.3720	± 0.00069	4.08	± 0.0061

2	9.20	± 0.54	0.3380	± 0.00019	17.01	± 0.54

0.024 mM

Slab #	**d** (Å)		**ρ** ($\frac{e}{Å^3}$)		**σ** (Å)	
1	23.64	± 0.55	0.3717	± 0.00063	4.08	± 0.0071
2	7.55	± 0.558	0.3386	± 0.00057	16.53	± 0.67

0.06 mM

Slab #	**d** (Å)		**ρ** ($\frac{e}{Å^3}$)		**σ** (Å)	
1	25.11	± 0.43	0.3712	± 0.00062	4.21	± 0.0051
2	6.94	± 0.54	0.3379	± 0.0005	15.79	± 0.48

0.12 mM

Slab #	**d** (Å)		**ρ** ($\frac{e}{Å^3}$)		**σ** (Å)	
1	26.23	± 0.140	0.3682	± 0.00030	4.34	± 0.0056
2	5.25	± 0.785	0.3381	± 0.00055	13.86	± 0.2096

Parameters: d is the layer thickness, ρ is the density, and σ is the interfacial roughness, uncertainties reflect MCMC of XRR fitted parameters.

Table S3. Fitting parameters from XRR measurements from adsorbed layers of solutions containing constant bulk concentration of polysorbate 80 (0.012 mM) and different bulk concentration of oleic acid.

0.012 mM PS80 + 0.017 mM OA

Slab #	**d** (Å)		**ρ** ($\frac{e}{Å^3}$)		**σ** (Å)	
1	12.60	± 0.259	0.2984	± 0.00103	4.08	± 0.0179
2	8.00	± 0.557	0.3873	± 0.00761	4.08	± 0.0179
3	17.17	± 0.322	0.3507	± 0.00032	4.08	± 0.0179

0.012 mM PS80 + 0.035 mM OA

Slab #	d (Å)		ρ ($\frac{e}{Å^3}$)		σ (Å)	
1	13.5	± 0.14	0.2979	± 0.0009	4.19	± 0.0166
2	7.00	± 0.35	0.3891	± 0.00245	4.19	± 0.0166
3	16.38	± 0.32	0.3480	± 0.00033	4.19	± 0.0166

0.012 mM PS80 + 0.071 mM OA

Slab #	d (Å)		ρ ($\frac{e}{Å^3}$)		σ (Å)	
1	13.03	± 0.11	0.2924	± 0.0008	4.176	± 0.0161
2	8.00	± 0.28	0.3874	± 0.00187	4.176	± 0.0161
3	17.99	± 0.301	0.3479	± 0.00025	4.176	± 0.0161

0.012 mM PS80 +0.106 mM OA

Slab #	d (Å)		ρ ($\frac{e}{Å^3}$)		σ (Å)	
1	13.02	± 0.27	0.2940	± 0.00194	4.218	± 0.037
2	9.5	± 0.59	0.3805	± 0.00282	4.218	± 0.037
3	16.88	± 0.32	0.3464	± 0.00039	4.218	± 0.037

Parameters: d is the layer thickness, ρ is the density, and σ is the interfacial roughness, uncertainties reflect MCMC of XRR fitted parameters.

Table S4. Fitting parameters from XRR measurements from adsorbed layers of solutions containing constant bulk concentration of polysorbate 80 (0.06 mM) and different bulk concentration of oleic acid.

0.06 mM PS80 + 0.017 mM OA

Slab #	d (Å)	ρ ($\frac{e}{Å^3}$)	σ (Å)

1	12.34	± 0.277	0.331	± 0.00102	4.35	± 0.0133
2	4.35	± 0.682	0.399	± 0.00291	4.35	± 0.0133
3	18.84	± 0.365	0.3506	± 0.00024	4.35	± 0.0133

0.06 mM PS80 + 0.035 mM OA

**Slab #**	**d** (Å)		$\boldsymbol{\rho}$ $(\frac{\mathbf{e}}{\text{Å}^3})$		$\boldsymbol{\sigma}$ (Å)	
1	12.8	± 0.21	0.3218	± 0.00118	4.44	± 0.0186
2	4.91	± 0.41	0.401	± 0.00377	4.44	± 0.0186
3	18.94	± 0.197	0.3505	± 0.00021	4.44	± 0.0186

0.06 mM PS80 + 0.071 mM OA

**Slab #**	**d** (Å)		$\boldsymbol{\rho}$ $(\frac{\mathbf{e}}{\text{Å}^3})$		$\boldsymbol{\sigma}$ (Å)	
1	13.02	± 0.17	0.3066	± 0.00112	4.406	± 0.0178
2	5.11	± 0.37	0.400	± 0.00410	4.406	± 0.0178
3	19.01	± 0.216	0.3511	± 0.00022	4.406	± 0.0178

0.06 mM PS80 + 0.106 mM OA

Slab #	**d** (Å)		$\boldsymbol{\rho}$ $(\frac{\mathbf{e}}{\text{Å}^3})$		$\boldsymbol{\sigma}$ (Å)	
1	13.32	± 0.164	0.3064	± 0.000102	4.413	± 0.0169
2	5.11	± 0.38	0.400	± 0.0037	4.413	± 0.0169
3	18.76	± 0.20	0.3497	± 0.0002	4.413	± 0.0169

Parameters: d is the layer thickness, ρ is the density, and σ is the interfacial roughness, uncertainties reflect MCMC of XRR fitted parameters.

Table S5. Fitting parameters from XRR measurements from adsorbed layers of solutions containing constant bulk concentration of polysorbate 80 (0.12 mM) and different bulk concentration of oleic acid.

0.12 mM PS80 + 0.017 mM OA

Slab #	**d** (Å)		**ρ** ($\frac{e}{Å^3}$)		**σ** (Å)	
1	10.82	± 0.076	0.333	± 0.00017	4.14	± 0.0099
2	7.00	± 0.060	0.3904	± 0.00064	4.14	± 0.0099
3	17.26	± 0.161	0.3567	± 0.00032	4.14	± 0.0099

0.12 mM PS80 + 0.035 mM OA

Slab #	**d** (Å)		**ρ** ($\frac{e}{Å^3}$)		**σ** (Å)	
1	11.16	± 0.14	0.333	± 0.00040	4.22	± 0.0119
2	7.50	± 0.37	0.3886	± 0.00173	4.22	± 0.0119
3	16.78	± 0.228	0.3555	± 0.00043	4.22	± 0.0119

0.12 mM PS80 + 0.071 mM OA

Slab #	**d** (Å)		**ρ** ($\frac{e}{Å^3}$)		**σ** (Å)	
1	12.56	± 0.25	0.3189	± 0.00122	4.386	± 0.0209
2	4.83	± 0.05	0.400	± 0.0048	4.386	± 0.0209
3	19.12	± 0.246	0.3498	± 0.0048	4.386	± 0.0209

0.12 mM PS80 + 0.106 mM OA

Slab #	***d*** (Å)		***ρ*** ($\frac{e}{Å^3}$)		***σ* (Å)**	
1	12.77	± 0.135	0.3101	± 0.00105	4.412	± 0.0177
2	5.33	± 0.24	0.4000	± 0.00465	4.412	± 0.0177
3	18.70	± 0.19	0.3504	± 0.00021	4.412	± 0.0177

Parameters: d is the layer thickness, ρ is the density, and σ is the interfacial roughness, uncertainties reflect MCMC of XRR fitted parameters.

Table S6. Summary of x-ray reflectivity measurements of surface concentrations for constant bulk concentration of polysorbate 80 at 0.06 mM and different bulk concentration of oleic acid.

$\frac{OA}{PS80}$ (bulk)	$\frac{OA}{PS80}$ (surface)	$\Gamma_{PS80}(\frac{mg}{m^2})$	$\Gamma_{OA}(\frac{mg}{m^2})$	$\Gamma_{W}(\frac{mg}{m^2})$
0	0	1.86 ± 0.63	0	1.66 ± 0.57
0.28	1.95 ± 1.73	1.48 ± 0.39	0.62 ± 0.52	1.64 ± 0.42
0.58	2.55 ± 1.17	1.41 ±0.25	0.78 ±0.33	1.63 ±0.26
1.18	3.89 ± 1.36	1.23 ±0.23	1.04 ±0.31	1.54 ±0.24
1.77	4.26 ± 1.46	1.19 ±0.23	1.09 ±0.31	1.52 ±0.25

Parameters: $\frac{OA}{PS80}$ is the molar ratio of OA and PS80, Γ_{PS80} is the surface concentration of PS80, Γ_{OA} is the surface concentration of oleic acid, Γ_{W} is the surface concentration of water, uncertainties reflect standard error propagation from MCMC-derived XRR fitted parameters.

Table S7. Summary of x-ray reflectivity measurements of surface concentrations for constant bulk concentration of Polysorbate 80 at 0.12 mM and different bulk concentration of oleic acid.

$\frac{OA}{PS80}$ (bulk)	$\frac{OA}{PS80}$ (surface)	$\Gamma_{PS80}(\frac{mg}{m^2})$	$\Gamma_{OA}(\frac{mg}{m^2})$	$\Gamma_{W}(\frac{mg}{m^2})$
0	0	1.78 ± 0.72	0	1.67 ± 0.65
0.14	0.86 ± 0.34	1.70 ± 0.09	0.32 ± 0.12	1.75 ± 0.10
0.29	0.97 ± 0.90	1.69 ±0.24	0.35 ±0.33	1.75 ±0.26
0.59	2.87 ± 1.49	1.36 ±0.30	0.84 ±0.40	1.60 ±0.32
0.88	3.47 ± 1.37	1.28 ±0.25	0.96 ±0.33	1.56 ±0.26

Parameters: $\frac{OA}{PS80}$ is the molar ratio of OA and PS80, Γ_{PS80} is the surface concentration of PS80, Γ_{OA} is the surface concentration of oleic acid, Γ_{W} is the surface concentration of water, uncertainties reflect standard error propagation from MCMC-derived XRR fitted parameters.

Dynamic light scattering

Figure S1 shows the dynamic light scattering (DLS) volume-weighted particle size distribution for 0.012 mM PS80, including the mean curve and standard deviation from four independent measurements. Figure S2 shows the corresponding DLS results for 0.012 mM PS80 mixed with oleic acid (OA), also reported as the mean distribution with standard deviation from four independent measurements.

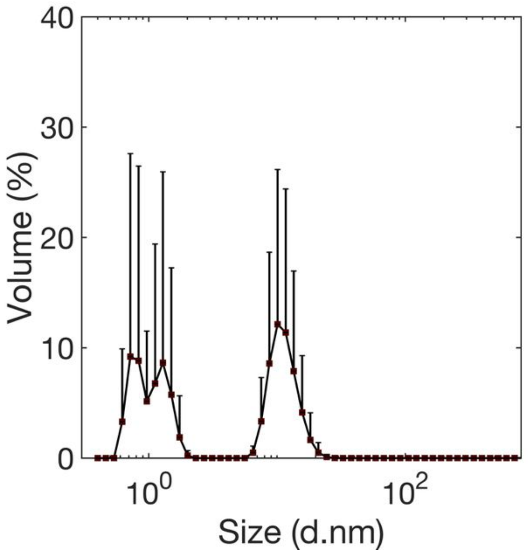

Figure S1. Dynamic light scattering volume distribution as a function of size for 0.012 mM PS80, error bars represent the standard deviation from four independent measurements.

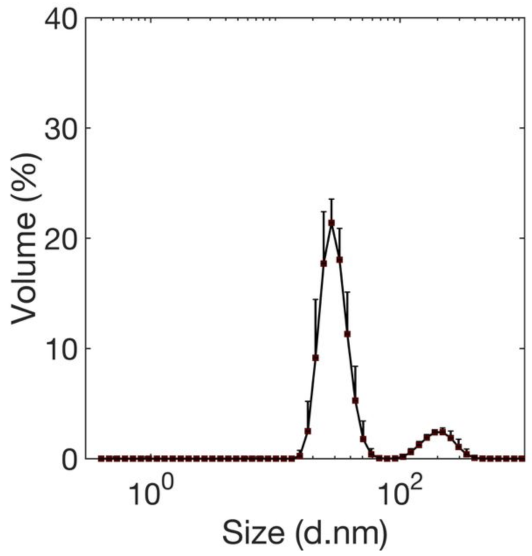

Figure S2. Dynamic light scattering volume distribution as a function of size for 0.012 mM PS80 in the presence of 0.106 mM OA, error bars represent the standard deviation from four independent measurements.

References

(1) Chang, C.-H.; Franses, E. I. Adsorption Dynamics of Surfactants at the Air/Water Interface: A Critical Review of Mathematical Models, Data, and Mechanisms. *Colloids Surf. Physicochem. Eng. Asp.* 1995, *100*, 1–45. https://doi.org/10.1016/0927-7757(94)03061-4.

(2) Kishore, R. S. K.; Kiese, S.; Fischer, S.; Pappenberger, A.; Grauschopf, U.; Mahler, H.-C. The Degradation of Polysorbates 20 and 80 and Its Potential Impact on the Stability of Biotherapeutics. *Pharm. Res.* 2011, *28* (5), 1194–1210. https://doi.org/10.1007/s11095-011-0385-x.

(3) Nagle, J. F.; Venable, R. M.; Maroclo-Kemmerling, E.; Tristram-Nagle, S.; Harper, P. E.; Pastor, R. W. Revisiting Volumes of Lipid Components in Bilayers. *J. Phys. Chem. B* 2019, *123* (12), 2697–2709. https://doi.org/10.1021/acs.jpcb.8b12010.

(4) Kučerka, N.; Nagle, J. F.; Sachs, J. N.; Feller, S. E.; Pencer, J.; Jackson, A.; Katsaras, J. Lipid Bilayer Structure Determined by the Simultaneous Analysis of Neutron and X-Ray Scattering Data. *Biophys. J.* 2008, *95* (5), 2356–2367. https://doi.org/10.1529/biophysj.108.132662.

(5) Tristram-Nagle, S.; Petrache, H. I.; Nagle, J. F. Structure and Interactions of Fully Hydrated Dioleoylphosphatidylcholine Bilayers. *Biophys. J.* 1998, *75* (2), 917–925. https://doi.org/10.1016/S0006-3495(98)77580-0.

(6) Høiland, H. Partial Molar Volumes of Biochemical Model Compounds in Aqueous Solution. In *Thermodynamic Data for Biochemistry and Biotechnology*; Hinz, H.-J., Ed.; Springer: Berlin, Heidelberg, 1986; pp 17–44. https://doi.org/10.1007/978-3-642-71114-5_2.
